# Causative Agents of American Tegumentary Leishmaniasis Are Able to Infect 3T3-L1 Adipocytes *In Vitro*

**DOI:** 10.3389/fcimb.2022.824494

**Published:** 2022-02-04

**Authors:** Bruno Mendes, Karen Minori, Silvio R. Consonni, Norma W. Andrews, Danilo C. Miguel

**Affiliations:** ^1^ Department of Animal Biology, Institute of Biology, State University of Campinas – UNICAMP, Campinas, Brazil; ^2^ Department of Biochemistry and Tissue Biology, Institute of Biology, State University of Campinas – UNICAMP, Campinas, Brazil; ^3^ Department of Cell Biology and Molecular Genetics, University of Maryland, College Park, MD, United States

**Keywords:** 3T3-L1, adipocyte, infection, *Leishmania*, microscopy

## Abstract

Although macrophages have long been considered key players in the course of *Leishmania* infections, other non-professional phagocytes have lately been shown to maintain low levels of the parasite in safe intracellular niches. Recently, it was demonstrated that the adipose tissue is capable of harboring Old World *L.* (*L.*) *infantum* in mice. However, there is no evidence of experimental adipocyte infection with New World *Leishmania* species so far. In addition, it was not known whether adipocytes would be permissive for formation of the unique, large and communal parasitophorous vacuoles that are typical of *L.* (*L.*) *amazonensis* in macrophages. Here we evaluated the ability of *L.* (*L.*) *amazonensis* and *L.* (*V.*) *braziliensis* promastigotes and amastigotes to infect 3T3-L1 fibroblast-derived adipocytes (3T3-Ad) using light and transmission electron microscopy. Our results indicate that amastigotes and promastigotes of both species were capable of infecting and surviving inside pre- and fully differentiated 3T3-Ad for up to 144 h. Importantly, *L.* (*L.*) *amazonensis* amastigotes resided in large communal parasitophorous vacuoles in pre-adipocytes, which appeared to be compressed between large lipid droplets in mature adipocytes. In parallel, individual *L. (V.) braziliensis* amastigotes were detected in single vacuoles 144 h post-infection. We conclude that 3T3-Ad may constitute an environment that supports low loads of viable parasites perhaps contributing to parasite maintenance, since amastigotes of both species recovered from these cells differentiated into replicative promastigotes. Our findings shed light on the potential of a new host cell model that can be relevant to the persistence of New World *Leishmania* species.

## Introduction

At least 20 species belonging to the *Leishmania* genus cause leishmaniasis, a complex disease with different clinical manifestations that leads to 20,000-30,000 annual deaths worldwide (Akhoundi et al., 2016; WHO, 2021). The disease has shown increased incidence and expansion of transmission to new territories in recent years (Pigott et al., 2014; Cotton, 2017).

Two main stages can be recognized throughout the life cycle of *Leishmania*: oval-shaped cells with an interiorized/reduced flagellum (amastigotes) and fusiform cells with a prominent flagellum (promastigotes). Amastigotes are taken up by female sand flies with the blood of an infected vertebrate and, upon reaching the digestive tract of the insect, transform into promastigotes that attach to the gut epithelium. After a few days, the parasites are released from the gut epithelium and accumulate in the insect’s stomodeal valve as non-replicative metacyclic promastigotes, prior to inoculation in the vertebrate host dermis where they will subvert innate defense mechanisms and infect mononuclear phagocytic cells, mainly macrophages. Within these cells, metacyclic promastigotes tolerate increase in temperature (~25 to ~34°C), decrease in pH and low iron availability, features directly responsible for triggering promastigote-to-amastigote differentiation inside phagolysosomal compartments (parasitophorous vacuoles, PVs) (Sacks and Kamhawi, 2001; Mittra et al., 2013). After transformation, amastigotes divide by binary fission several times and rupture the host cell. Finally, the mononuclear phagocyte system will internalize these newly released parasites both locally and after dissemination, leading to the classical leishmaniasis symptoms (McCall et al., 2013).

Infected patients can develop the cutaneous form of leishmaniasis that affects skin, lymph nodes and mucous membranes, or the visceral form, with spleen, bone marrow and liver parasitism. Disease treatment is challenging due to high cost, drug toxicity, hospitalization need for parenteral drug administration, and variable efficacy that can lead to disease recurrence. In addition, reports of parasite resistance to antimonials, used as the first drug choice in many regions, have been a matter of concern in the context of antileishmanial chemotherapy (Darcis et al., 2017; Souza et al., 2017; Alcântara et al., 2018).

The cellular and molecular mechanisms underlying the chronicity of leishmaniasis, common in relapsing patients, are not fully understood. However, a few studies revealed that hyperactivation of the inflammatory response mediated by *Leishmania* plays an important role during the establishment of chronic infections (Navas et al., 2014). Furthermore, distinct cell types, such as fibroblasts and hepatocytes, may be involved in disease control and spread of the parasite to different tissues (Bogdan et al., 2000; Morehead et al., 2002; Vianna et al., 2002; Cavalcante-Costa et al., 2019). Adipocytes, for example, are not only storage cells within tissues and organs, but are also associated with inflammatory signaling in metabolic diseases (Cristancho and Lazar, 2011). Interestingly, Allahverdiyev and co-authors described experimental infections of adipocytes derived from mesenchymal stem cells with the following Old World *Leishmania* species: *Leishmania (Leishmania) donovani*, *L. (L.) major*, *L. (L.) tropica*, and *L. (L.) infantum* (Allahverdiyev et al., 2011). Also, a very recent study showed that murine adipose tissue harbors Old World *L. (L.) infantum* (Schwing et al., 2021).

There is no evidence of experimental adipocytic infection with New World *Leishmania* species in the literature so far. However, given the implication of adipocytes in inflammatory signaling and recent evidence of successful *in vitro* and *in vivo* infection by Old World *Leishmania* species, it is reasonable to speculate that the establishment of adipocytic infections with *Leishmania* species that cause American Tegumentary Leishmaniasis (ATL), i.e., *L.* (*L.*) *amazonensis* and *L.* (*Viannia*) *braziliensis*, may not only be feasible, but also play an important role in parasite persistence. In fact, in addition to the pathologies being quite distinct in the regions where both species circulate, there are also differences related to the formation and organization of the intravacuolar environment occupied by species of the *Leishmania mexicana* complex (i.e., *L. (L.) amazonensis*) when compared with other species. Taken together, all aspects above-mentioned highlight the importance of knowing this new model of infection by *Leishmania* spp.

## Methods

### Parasite Cultivation

*Leishmania (L.) amazonensis* (IFLA/BR/67/PH8 WT and GFP strains (Renberg et al., 2015)) and *Leishmania (V.) braziliensis* (MHOM/BR/75/2903 strain) promastigotes were cultivated in medium 199 with 20% heat-inactivated fetal bovine serum (FBS), 10 mM adenine, 5 mM L-glutamine, penicillin (100 U/mL) and streptomycin (100 μg/mL) (Sigma-Aldrich, Merck KGaA, USA) in 25 cm^2^ cell culture flasks at 25°C. Cultures of *L. (V.) braziliensis* were supplemented with 10% FBS and 5% sterile male human urine. *L. (L.) amazonensis* and *L. (V.) braziliensis* amastigotes were cultivated axenically as previously described (Miguel et al., 2013) in modified medium 199 supplemented with 20% FBS acidified to pH 4.8 and pH 5.2, respectively. Passages were kept for up to two weeks at 32°C for *L. (L.) amazonensis* and 34°C for *L. (V.) braziliensis*.

### Differentiation of 3T3-L1 Fibroblasts

3T3-L1 fibroblasts were grown in DMEM medium (Gibco, Thermo Fisher) supplemented with 10% FBS, 2 mM L-glutamine, 0.1 M sodium pyruvate, 40 mM HEPES and 1% penicillin (100 U/mL) and streptomycin (100 µg/mL) (Sigma-Aldrich) in 25 cm^2^ culture vented flasks at 37°C with 5% CO_2_. The culture was split every three days from 10^5^ cells/mL. 3T3-L1 fibroblast-derived adipocytes (3T3-Ad) were obtained as previously described (Zebisch et al., 2012), with some modifications. The total period of cell differentiation consisted of 14 days, with medium replacement every 48 h. Initially, 10^4^ cells/mL were seeded in 24-well plates (Corning) containing 13 mm coverslips. On day 3, cultures at 70-80% of confluence were exposed to the adipogenic cocktail containing 0.25 mM dexamethasone, 2 µg/mL insulin and 0.5 mM IBMX (Sigma-Aldrich) for 48h at 37°C, 5% CO_2_. On day 5, the supernatant was replaced by fresh medium containing insulin at 1 µg/mL for additional 48 h. On days 7-14, medium replacements were performed every 48 h. Pre-adipocytes containing small lipid droplets and a few mature 3T3-Ad were detected from day 8. Mature 3T3-Ad were observed until day 14.

### *In Vitro* Infections and Staining Protocols

Pre- and mature 3T3-Ad were infected with *Leishmania* late stationary phase-promastigotes or axenic amastigotes (multiplicity of infection (MOI) = 20) in 24-well plates containing 13 mm glass coverslips and kept at 34°C with 5% CO_2_. After 4 h of incubation, wells were washed three times with warm PBS (1X) and incubated for 1, 24, 48 and 144h at 34°C with 5% CO_2_. Next, coverslips were washed twice with warm PBS (1X) and fixed in 4% formaldehyde solution for 1h. Microscopic examination of unstained cells was performed using EVOS imaging systems (ThermoFisher Scientific). In parallel, cultures were sequentially stained as follows: one part Oil-Red O stock solution in ddH_2_O Zebisch et al. (2012) (2:1; v:v) for 2h, washed three times with ddH2O and then incubated with Giemsa solution for 15 min. Next, coverslips were gently rinsed in water. Intracellular parasite counts were obtained by quantifying the parasite number for at least 100 3T3-Ad per condition under a light microscope (100x oil immersion objective, Eclipse E200, Nikon).

For BODIPY lipid staining, coverslips were washed twice with warm PBS (1X) and fixed with 4% paraformaldehyde for 30 min at room temperature. Following fixation, samples were washed as describe above and permeabilized with 0.5% Triton for 20 min. Next, coverslips were incubated with 1μg/mL BODIPY 493/503 (Invitrogen) for 20 min. Samples were washed and mounted with Prolong Gold Antifade With Dapi (Invitrogen) and observed with the Zeiss Axio Imager 2 epifluorescence microscope. Images were processed using ImageJ 1.50b software (NIH USA).

For infections with *L*. (*L*.) *amazonensis* expressing GFP, 3T3-Ad were incubated with axenic amastigotes (MOI=20) in 24-well plates containing 13 mm glass coverslips and kept as described above. Coverslips were washed in warm PBS (1X) and carefully mounted onto microscope slides for examination of GFP amastigotes (Ex/Em 488/510 nm) using a Zeiss Axiovert 135 microscope equipped with digital camera (Orca II, Hamamatsu) controlled by Metamorph software (Universal Imaging). The tests were carried out in triplicates and with at least two independent experiments. Statistical analyzes were performed with Origin8 software (OriginLab) using Student’s t-test, with differences considered significant when p-value < 0.05.

### Transmission Electron Microscopy

Samples of 3T3-Ad infected with *Leishmania* were washed three times with warm PBS (1X), fixed with 2.5% glutaraldehyde in 0.1M sodium cacodylate buffer and 3 mM CaCl2 pH 7.4 for 5 min at room temperature and 1h at 4°C. The samples were washed three times with 0.1M sodium cacodylate buffer and 3mM CaCl2 at 4°C and three times with 0.1 M imidazole buffer pH 7.4 at 4°C to produce prominent electron-opaque staining of lipid droplets. Next, the cells were post-fixed with 2% osmium tetroxide in 0.1 M imidazole buffer pH 7.4, for 30 minutes at 4°C. Next, after five washes with ddH2O water at 4°C, samples were treated with filtered 2% aqueous uranyl acetate overnight at 4°C. After this, five washes with ddH2O water at 4°C was performed and sequential dehydration with ethanol was done at 4°C. Next, samples were embedded in Epon resin mixed with ethanol (1:1) under constant agitation for 30 min at room temperature. Embedding with pure resin was repeated five times. Samples were included in pure Epon resin for 72h at 60°C for complete polymerization. Finally, the material was sectioned and contrasted with 2% aqueous uranyl citrate for 20 min and lead citrate for 10 min at room temperature. Transmission electron microscopy analysis was performed using a LEO 906 Microscope (Zeiss) at the Electron Microscopy Laboratory (Institute of Biology, UNICAMP).

### Assessment of Amastigote Viability

Amastigotes’ viability was assessed by monitoring the transformation of intracellular parasites into promastigotes after 144 h of *in vitro* infection. For this, infected 3T3-Ad cultures were lysed with 0.04% SDS (sodium dodecyl sulfate) in 300 µL PBS (1X) through 10 cycles of resuspension of the content using a 30-G needle in 1 mL sterile syringes. The suspension was collected and washed with alternate cycles of centrifugation. First, suspensions were centrifuged at 100 x g for 10 minutes at 4°C for recovering supernatants containing amastigotes. Next, samples were resuspended in cold PBS (1X) and centrifuged at 800 x g for 10 minutes at 4°C to remove supernatants, while pellets were resuspended in PBS (1X). This last cycle was repeated twice. Pellets containing amastigotes were seeded in 96-well plates (10^5^/mL) with M199 medium for promastigote differentiation at 25°C for up to 9 days. Growth curves after differentiation of viable cells were determined by counting the number of promastigotes with a Neubauer chamber every other day.

## Results

**Figure 1:**
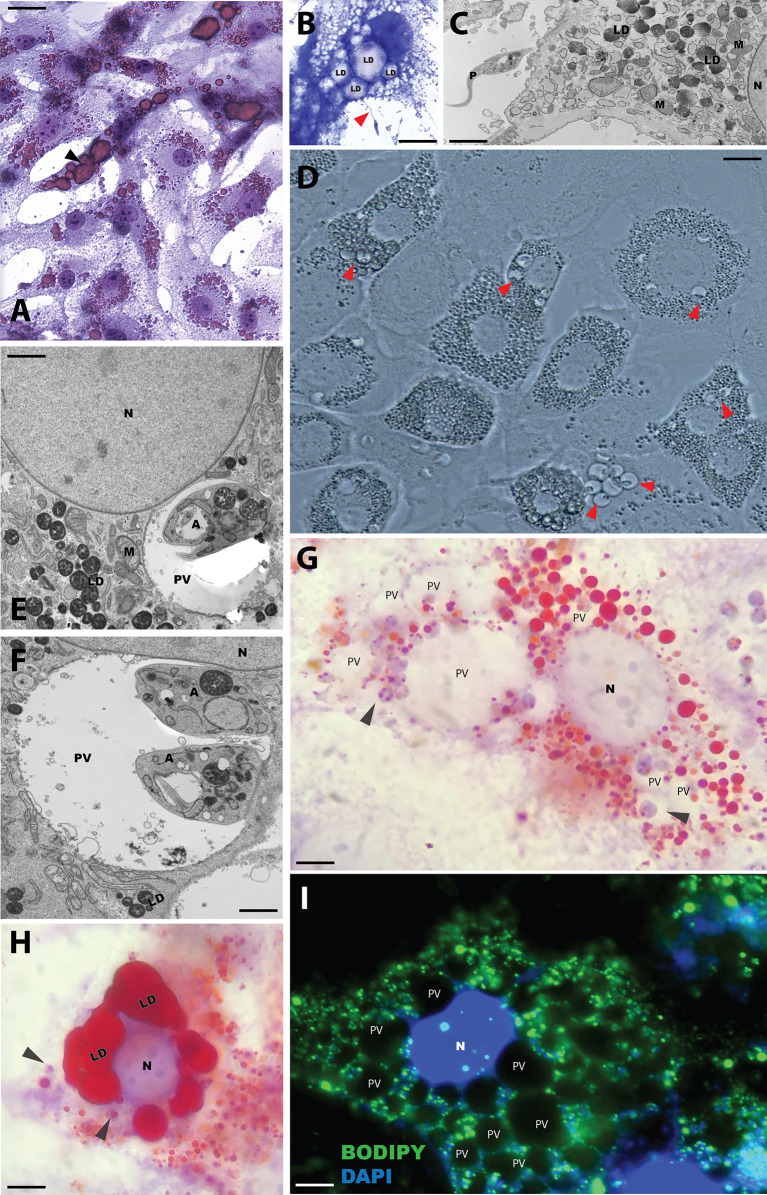
Representative micrographs of 3T3-Ad *in vitro* infected with *L. (L.) amazonensis*. **(A)** 3T3-L1 fibroblasts undergoing adipocyte differentiation after 14 days, in which lipid droplets are stained with Oil-Red O (arrowhead) and are observed in differentiated adipocytes stained simultaneously with Giemsa. Scale bar = 8 µm. **(B)** Differentiated adipocyte stained with Giemsa, in which hyaline lipid droplets (LD) spaces can be seen, were incubated with *L. (L.) amazonensis* stationary phase promastigotes (MOI=20) for 1 h. Note the promastigote attached to the adipocyte by the flagellum (red arrowhead). Scale bar = 12 µm. **(C)** Ultrastructure showed a promastigote (“P”) close to the plasma membrane of an 3T3-Ad. “M”: mitochondria, “LD”: electron-opaque lipid droplets after osmium-imidazole treatment, “N”: nucleus. Scale bar = 5 µm. **(D)** Unstained infected 3T3-Ad as described in **(B)**, observed by phase contrast microscopy after 48 h. Red arrows point to PVs containing amastigotes. Scale bar = 6 µm. **(E, F)** Ultrastructure of PV with amastigotes **(A)** infected for 48h “M”: mitochondria, “LD”: electron-opaque lipid droplets after osmium-imidazole treatment, “N”: nucleus. Scale bar = 0.5 µm. Representative 3T3-L1 pre-adipocyte **(G)** and 3T3-Ad **(H)** infected for 48 h and stained with Giemsa and Oil-Red O, where small red droplets (LD) are dispersed throughout the cytosol close to PVs harboring amastigotes (arrowheads) **(G)**. Bulky droplets (LD) stained in red **(H)**, with amastigotes nearby (arrowheads). “N”: nucleus. Scale bars **(G)** = 2.5 µm; **(H)** = 3.5 µm. **(I)** Fluorescence microscopy of infected 3T3-Ad for 48 h. Lipid droplets stained with BODIPY 493/503 (green) are closely seen with PV. “N”: nucleus of host cell and parasites stained in blue with DAPI. Scale bar = 6 µm.

Adipocyte cultures were established by incubation of 3T3-L1 fibroblasts with the differentiation cocktail for 8 days. With this protocol we reached a differentiation rate of approximately 70-80% and several cells containing Oil-Red O-stained lipid droplets were easily identified by Giemsa staining and light microscopy (**Figure 1A**). Differentiated cultures (referred to as 3T3-Ad) were incubated with *L. (L.) amazonensis* promastigotes for 4 h and various staining protocols were tested to determine the best fixation/dye method that allowed proper visualization of *Leishmania* and 3T3-Ad interactions. Contact of the flagellar tips of promastigotes with 3T3-Ad was detected in methanol-fixed preparations stained with Giemsa, where lipid droplets were visualized as hyaline spaces (**Figure 1B**), and also by live light microscopy imaging (**Video 1; Supplementary Material**). Promastigotes close to 3T3-Ad were also seen by transmission electron microscopy (**Figure 1C**).

Internalized *L. (L.) amazonensis* promastigotes typically differentiated into amastigotes after 48 h, and occupied large PVs surrounded by lipid droplets (**Figures 1D–F**). Clear visualization of PVs interspersed with lipid droplets in infected pre-adipocytes was possible using the Giemsa and Oil-Red O co-staining protocol (**Figure 1G**). However, in fully differentiated 3T3-Ad, that contains larger lipid droplets, observation of the parasites was challenging and depended on the focal position (**Figure 1H**). *L. (L.) amazonensis*-infected 3T3-Ad were also examined by fluorescence microscopy after staining the lipid content with BODIPY 493/503 (green) (**Figure 1I**). Undoubtedly, these photomicrographs clearly evidenced BODIPY staining in bodies close to amastigote-containing PVs (**Figure 1I**).

**Figure 2:**
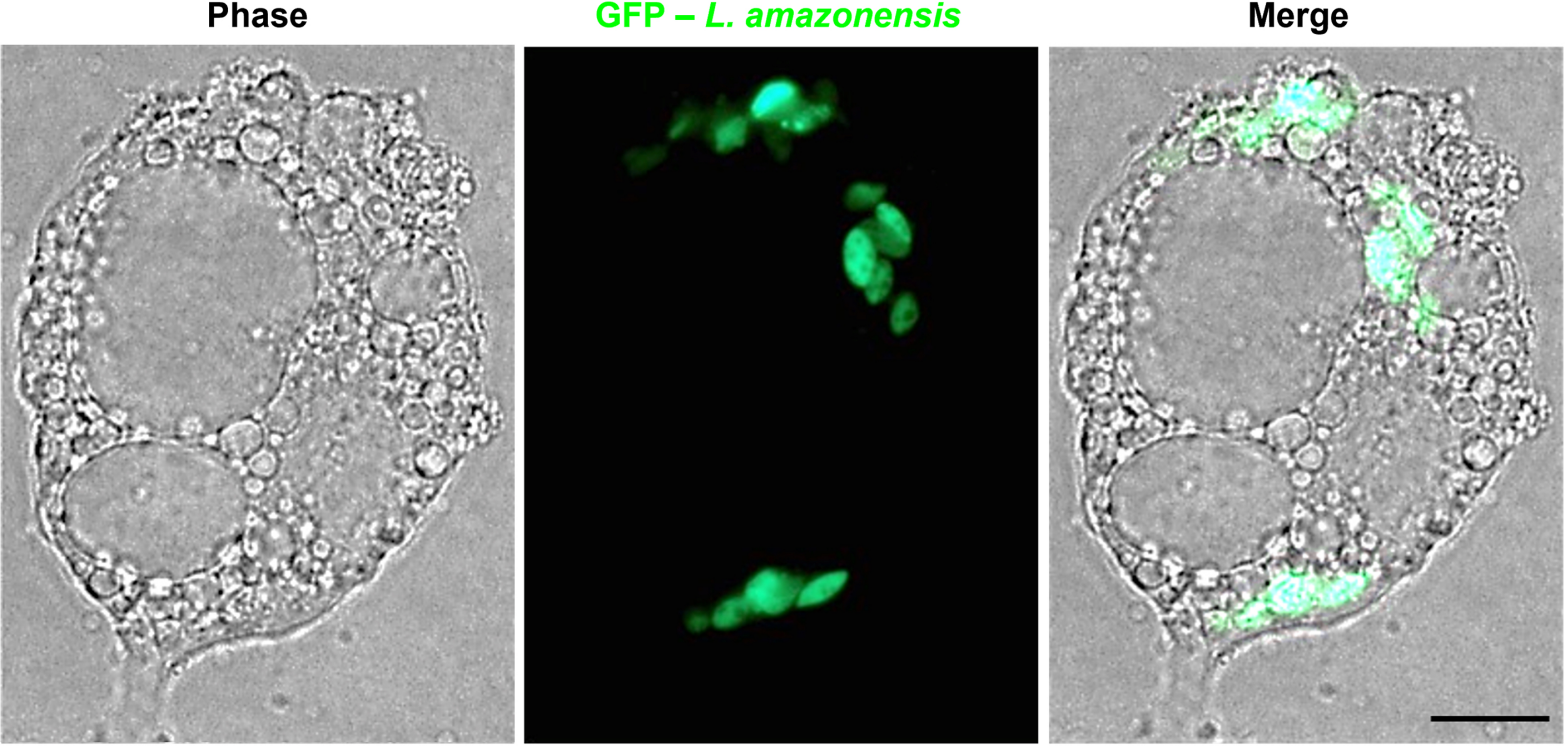
Unfixed 3T3-Ad infected with GFP – *L. (L.) amazonensis* for 144 h. Fully-differentiated 3T3-Ad showing intracellular amastigotes expressing GFP (green) accumulated among lipid droplets. Scale bar = 5 µm.

To avoid possible deformation of lipid droplets caused by staining protocols and to more precisely determine the intracellular distribution of parasites, assays were conducted in live cells with green fluorescent amastigotes (GFP – *L. amazonensis*). **Figure 2** shows that after 144 h, amastigotes internalized in 3T3-Ad were tightly localized between large and abundant lipid droplets.

**Figure 3:**
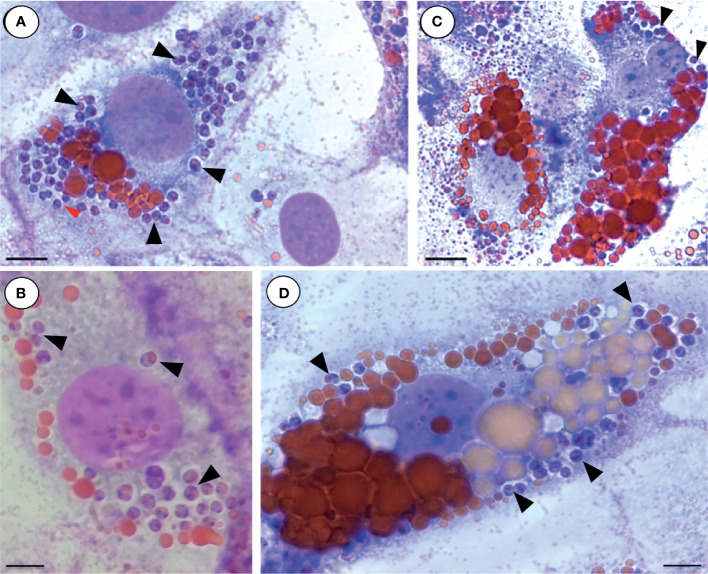
Infection of 3T3-Ad with *L. (V.) braziliensis*. 3T3-Ad presenting reduced **(A, B)** or increased number of lipid droplets **(C, D)** were infected with axenic amastigotes of *L. (V.) braziliensis* for 24 h (MOI=20) and stained with Giemsa (purple nuclei and purple amastigotes) and Oil-Red O (lipid droplets in red). Arrowheads point to single amastigotes and individual PVs. Bars **(A)** = 6 µm; **(B, D)** = 5 µm, **(C)** = 8 µm.

Taken together, our results indicate that the expanded and communal PVs typical of *L. (L.) amazonensis*-infected macrophages also form in non-phagocytic cells containing lipid droplets O (**Figures 1E–G**). However, fully differentiated adipocytes imposed visual challenges due to possible compression of the large communal PVs by the large lipid droplets. The typical morphology for *L. (V.) braziliensis* PVs in macrophage infections, i.e., single and tight vacuoles harboring the amastigote stage, was also observed for 3T3-Ad containing reduced or increased number of lipid droplets (**Figure 3**).

**Figure 4:**
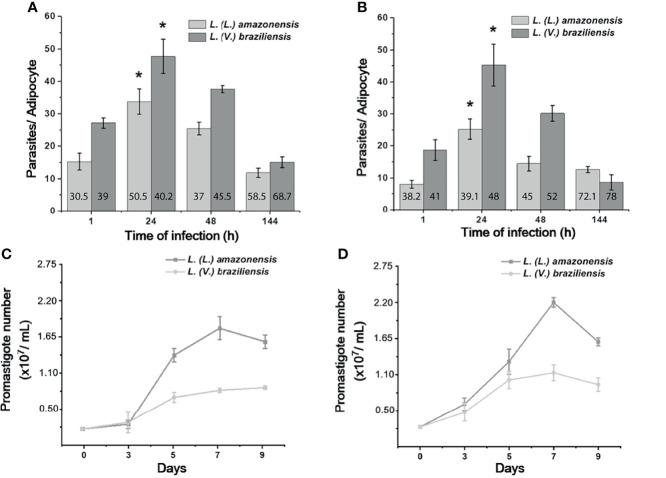
*In vitro* kinetics of *Leishmania* intracellular growth after 3T3-Ad infection, and parasite viability. The mean number of amastigotes per 3T3-Ad ± standard deviation was determined after infection with promastigotes **(A)** or axenic amastigotes **(B)** and incubation for 1, 24, 48 and 144 h, for *L. (L.) amazonensis* and *L. (V.) braziliensis*. At least 100 3T3-Ad cells were counted per condition. Assays were performed in triplicate and the assays were repeated at least twice. Asterisks indicate a statistically significant difference (*p*-value < 0.05) for each time point *vs*. 1 h (Student’s *t* test). Numbers in the bars indicate the mean of infected 3T3-Ad (%) per condition. **(C, D)**: Growth curves of parasites recovered from 3T3-Ad infections with promastigotes or amastigotes, respectively, after 144 h. Amastigote-promastigote differentiation was carried out as described in the ‘Methods Section’ and recovered parasites resuspended in M199 were kept at 25°C. On the 3rd day, promastigotes were observed, which grew exponentially until reaching the maximum density peaks around day 7. Experiments represent the mean of triplicates ± standard deviation.

Once the presence of the parasite was confirmed in our cell model, our next step was to investigate whether 3T3-Ad were able to sustain viable *L.* (*L.*) *amazonensis* and *L.* (*V.*) *braziliensis* intracellular parasites. Both stages were able to infect and persist in 3T3-Ad for up to 144 h, despite the detection of fewer amastigotes after 48 h (**Figures 4A, B**). Intracellular parasites were recovered and *in vitro* promastigote differentiation assays were performed, followed by assessment of the parasite’s ability to replicate by counting the cells over a period of 9 days (**Figures 4C, D**). Our results indicate that amastigotes of both *Leishmania* species remained viable in 3T3-Ad infected for 144 h, being capable of differentiating into promastigotes and replicating in culture.

## Discussion

Mononuclear phagocytes are recognized as the main target cells for parasitism by *Leishmania* amastigotes. However, a number of cell types have been suggested as potential additional infection sites by *Leishmania* (Rittig and Bogdan, 2000), including adipocytes (Allahverdiyev et al., 2011; Schwing et al., 2021). Studies concerning the adipose tissue have been limited to morphofunctional aspects for many decades due to its putative stable and immutable nature. However, this paradigm has been revisited with an increasing number of studies correlating this tissue to cardiovascular diseases, diabetes and inflammatory modulation (Cristancho and Lazar, 2011; Groeneveld, 2020). Moreover, evidence has been accumulating indicating that the adipose tissue may play a role in infections caused by bacteria, virus and protozoan parasites, perhaps by providing nutrients to allow the pathogen’s proliferation and survival (Shoemaker et al., 1970; Desruisseaux et al., 2007; Tanowitz et al., 2017; Bouzid et al., 2017; Bourgeois et al., 2019).

Concerning the ‘trypanosomatid-adipocyte’ interaction, it has been reported that *Trypanosoma brucei* and *T. cruzi* can associate with mouse white and brown adipose tissues, respectively (Shoemaker et al., 1970; Trindade et al., 2016). However, replicative forms of *T. brucei* are extracellular and, for *T. cruzi*, nucleated host cells are susceptible to active invasion and colonization by its replicative forms. With regard to the biology of *Leishmania* infection, its preference for phagocytic cells, particularly macrophages, has been very clear and considered to be directly associated with the development of leishmaniasis (Novais et al., 2014).

Leishmaniasis is classically manifested in its cutaneous or visceral form, affecting skin layers and internal organs, respectively. In both cases, adipocytes should be found occupying interstitial compartments, suggesting that *Leishmania* may interact with adipocytes in the event of both cutaneous and visceral leishmaniasis. Based on this hypothesis and on the fact that there is no evidence in the literature showing that ATL-causing species are capable to infect adipocytes, we investigated the ability of promastigotes and amastigotes of *L. (L.) amazonensis* and *L. (V.) braziliensis* to infect and replicate in 3T3-Ad.

Several specific staining protocols and microscopic techniques were employed in our study to confirm the adipocytes’ permissiveness to *Leishmania*. The panel in **Figure 1** illustrates the infective process of *L. (L.) amazonensis* in 3T3-Ad by revealing the interaction of promastigotes *via* flagellar tip (**Video 1; Supplementary Material**) and the presence of intracellular amastigotes at longer times of infection. When macrophages are infected by *L. (L.) amazonensis*, unique expanded and communal PVs containing several amastigotes are observed (Real et al., 2010) and, in our assays, large communal PVs were also detected in 3T3-Ad containing *L. (L.) amazonensis*, especially in host cells with low amounts or smaller sizes of lipid droplets (i.e., pre-adipocytes) (**Figure 1G, I**). In fact, the 3T3-L1-differentiation process is considered a dynamic process, as it promotes droplet remodeling during lipolysis and cell growth. Paar et al. showed that the growth of lipid droplets during human adipose-derived stem cells differentiation into adipocytes is equivalent to the process observed in 3T3-L1 cells (Paar et al., 2012). Our 3T3-Ad reached full differentiation as determined by the presence of larger droplets, and although this made it more challenging to observe the limits of PVs, it was clear that these cells contained viable amastigotes (**Figures 1H**, **2**). With regard to *L. (V.) braziliensis* infection, Giemsa and Oil-Red O-stained cells showed intact individualized PVs containing single amastigotes (**Figure 3**), as expected for this species in macrophage infections. Thus, an important finding of our study is that it clearly indicates that the dynamics of generation and maintenance of the adipocytic vacuolar environment, which is very different between *L. (L.) amazonensis* and *L. (V.) braziliensis*, is controlled by the parasite itself, not depending on unique characteristics of phagocytic host cells, as it has been previously shown that fibroblasts are capable of sustaining *Leishmania* infections (Morehead et al., 2002; Wilson et al., 2008).

ATL caused by *L. (L.) amazonensis* and *L. (V.) braziliensis* show different lesion aspects with inflammatory infiltrates that vary in their major cellular components (de Souza et al., 2011; de Oliveira and Brodskyn, 2012). However, phagocytic cells usually reveal the communal PV architecture greatly altered when filled with *L. (L.) amazonensis* amastigotes. This histopathological modulation of the vacuolar environment seems to be a strategy for parasite survival, as it may lead to the dilution of molecules with leishmanicidal action (Sacks and Sher, 2002; Wilson et al., 2008).

Quantification of the parasite burden revealed that promastigotes and axenic amastigotes had a similar capacity to infect 3T3-Ad, with infection levels peaking after 24 h for both species. Moreover, amastigotes of *L. (L.) amazonensis* and *L. (V.) braziliensis* were viable intracellularly up to after 144 h, as parasites were able to differentiate into flagellate promastigote forms, reproduce asexually and reach maximum density in culture growth curves (**Figure 4**). It is interesting to point out that infections with reduced MOI (2 and 5) were not efficient in sustaining *L. (V.) braziliensis* infections in our preliminary assays (data not shown). During the establishment of our experimental conditions, we observed that the most comparable infection rates were found using MOI=20 for both species. Perhaps differential infectivity potential presented by distinct strains from different species explain the success related to the ability of infecting adipocytes. These parameters should be considered in future studies, especially considering that ATL caused by *L. (L.) amazonensis* usually show substantial parasite burden while *L. (V.) braziliensis* infection leads to infiltrates with scarce amastigotes (Pereira et al., 2017). In this sense, adipocytes could play a distinct role in parasite maintenance that deserves to be investigated in depth in terms of species specificity.

Our findings with an easily accessible cell line open avenues for investigating whether adipose cells represent a suitable environment for *Leishmania* survival, especially related to its dependence on lipid metabolism/metabolites to survive in the host (Saunders and McConville, 2020), and what is the possible impact(s) of residence in adipose tissues for the pathogenesis of the disease. In addition, our results showed for the first time that New World *Leishmania* species that are causative agents of ATL are able to infect adipocytes *in vitro*, and maintain their ability to generate markedly distinct PVs in non-phagocytic cells. This unexplored aspect of the *Leishmania*-host interaction can be of great value for a deeper investigation of chronic cases, which are generally related to ineffective treatment regimens or natural relapses of ATL.

Furthermore, it has been shown that *Leishmania* persistence plays a relevant role in parasite dissemination, especially for New World species (Ramirez and Guevara, 1997; Soliman, 2006). In this sense, one of the most intriguing issues surrounding the pathogenesis of different clinical forms of leishmaniasis concerns the characterization of hideouts that serve as refuges for amastigotes to reproduce slowly. Adipocytes should be further investigated as possible sites for *Leishmania* persistence as they are capable of producing nitric oxide (NO), although at lower rates than macrophages (Pilon et al., 2000). Since the longstanding expression of inducible NO synthase is related to the persistence of *Leishmania* within host macrophages (Bogdan et al., 1996), it is plausible to suggest that another cell type involved in the infection course could allow the maintenance of this pathogen in certain organs where adipocytes are abundant, such as skin and viscera, which are relevant to specific clinical manifestations of different *Leishmania* species.

## Data Availability Statement

The raw data supporting the conclusions of this article will be made available by the authors, without undue reservation.

## Author Contributions

NA and DM: conceptualization of the study. BM, KM, SC, and DM: acquisition, analysis, and interpretation of the data. DM: writing - original draft preparation. BM, KM, SC, NA, and DM: writing - review and editing. SC, NA, and DM: supervision, project administration and funding acquisition. All authors contributed to the article and approved the submitted version.

## Funding

This work was supported by FAPESP [#14/21129-4] and Pró-Reitoria de Pesquisa - UNICAMP [#519.292] to DM, by CNPq [#421841/2018-4] and FAPESP [#2017/21720-2] to SC and by National Institutes of Health [grant RO1 AI067979] to NA. BM and KM received CAPES-DS and FAPESP [#15/17902-2] scholarships, respectively.

## Conflict of Interest

The authors declare that the research was conducted in the absence of any commercial or financial relationships that could be construed as a potential conflict of interest.

## Publisher’s Note

All claims expressed in this article are solely those of the authors and do not necessarily represent those of their affiliated organizations, or those of the publisher, the editors and the reviewers. Any product that may be evaluated in this article, or claim that may be made by its manufacturer, is not guaranteed or endorsed by the publisher.
